# Supplementary material for: Spatial eco-evolutionary feedbacks mediate coexistence in prey-predator systems
Source: Sci Rep. 2019 Dec 3;9:18161. doi: 10.1038/s41598-019-54510-6 (PMC6890681; doi:10.1038/s41598-019-54510-6)
Supplement: Supplementary file 3 — Supplementary material [file 41598_2019_54510_MOESM3_ESM.pdf]

Supplementary material for: "Spatial eco-evolutionary feedbacks mediate coexistence in prey-predator systems"

Eduardo H. Colombo,[1,*] Ricardo Martínez-García,[2] Cristóbal López,[1] and Emilio Hernández-García[1]

[1]IFISC (CSIC-UIB), Campus Universitat Illes Balears, 07122, Palma de Mallorca, Spain
[2]Department of Ecology and Evolutionary Biology, Princeton University, Princeton NJ 08544, USA

I. THE DYNAMICS OF THE DISTRIBUTION OF PERCEPTUAL RANGES IN THE WELL-MIXED LIMIT

In this section, we consider the dynamics of the distribution of predators' perceptual ranges, $\rho(R,t)$, in the well-mixed case. The normalization of this distribution is defined such that, at each time, the total number of predators is given by $N_p = \int dR\, \rho(R,t)$. On average, the encounters between prey and predators lead to an expected rate of change of $\rho(R)$, $\widetilde{\rho(R)}$, given by

$$\widetilde{\rho(R)} = b\rho(R)\langle c(R)\rangle_p \,, \tag{S1}$$

which is proportional to the mean predation rate $\langle c(R)\rangle_p$ (averaged over all predators that experience different environments but with the same R) multiplied by the probability b of birth after a predation event.

In a spatially homogeneous situation, and if the number of individuals is large enough so that we can neglect demographic fluctuations, the expected number of prey individuals within a radius R is $\langle M_v(R)\rangle_p = \pi R^2 v$, where v is the (uniform) density of prey. Thus $\langle c(R)\rangle_p = \langle E(R) M_v(R)\rangle_p = c_0 \pi R^2 v e^{-R/R_c}$ (see Methods in the main text for further details). Next, because mutations change the perceptual ranges of the new individuals as compared to that of their parents, and adding the contribution from the predator death at fixed rate d, the distribution of perceptual ranges evolves according to

$$\begin{aligned} \frac{\partial \rho(R,t)}{\partial t} &= \int_0^{L/2} G_\mu(R,R')\widetilde{\rho(R')}dR' - d\rho(R) \\ &= \pi b c_0 v \int_0^{L/2} G_\mu(R,R')\rho(R')(R')^2 e^{-R'/R_c} dR' - d\rho(R)\;. \end{aligned} \tag{S2}$$

The density of prey, on the other hand, changes following

$$\begin{aligned} \frac{dv}{dt} &= rv - \int_0^{L/2} \langle c(R)\rangle_p \rho(R) dR\,, \\ &= rv - \pi c_0 v \int_0^{L/2} R^2 e^{-R/R_c}\rho(R)dR\,, \end{aligned} \tag{S3}$$

where the first term represents prey birth at constant rate r and the second one accounts for predation.

The integral kernel G_μ relates newborn with parental perceptual ranges through mutations that are random perturbations to the parental perceptual range that follow a Gaussian distribution of zero mean and standard deviation σ_μ. The kernel G_μ should also account for boundary conditions in R, such that mutations leading to perceptual ranges that are either negative or larger than half of the system size are rejected. Thus, G_μ is a truncated Gaussian function in the interval $[0, L/2]$,

$$G_\mu(R,R') = \begin{cases} \frac{1}{\mathcal{N}(R)} e^{\frac{-(R-R')^2}{2\sigma_\mu^2}} & 0 < R' < L/2 \\ 0 & \text{else} \end{cases}\,, \tag{S4}$$

*Electronic address: ecolombo@ifisc.uib-csic.es

where $\mathcal{N}$ is a normalization factor given by $\mathcal{N}(R) = \sqrt{\frac{\pi}{2}}\sigma_\mu \left[\text{erf}\left(\frac{R}{\sqrt{2}\sigma_\mu}\right) - \text{erf}\left(\frac{R-L/2}{\sqrt{2}\sigma_\mu}\right)\right]$.

Lastly, note that Eq. (S3), together with Eq. (S2) integrated with respect to R to obtain the dynamics of N_p, recover the classical Lotka-Volterra predator-prey equations (with a predation rate that depends on R^*) in the limit of vanishing trait variability, $\rho(R,t) \to N_p\delta(R - R^\star)$.

II. SUPPLEMENTARY MOVIES AND FIGURES

A. Movies

[S1_movie.mp4 on electronic supplementary material]

S1 Movie **Low diffusion. Temporal evolution of the spatial distribution of the prey and predator populations.** Predator and prey individuals are represented by red and blue symbols respectively. Parameter values: $D_p = D_v = 1$, mutation intensity $\sigma_\mu = 0.1$ and habitat size $L = 20$.

[S2_movie.mp4 on electronic supplementary material]

S2 Movie **High diffusion. Temporal evolution of the spatial distribution of the prey and predator populations.** Predator and prey individuals are represented by red and blue symbols respectively. Parameter values: $D_p = D_v = 1$, mutation intensity $\sigma_\mu = 0.1$ and habitat size $L = 20$.

B. Figures

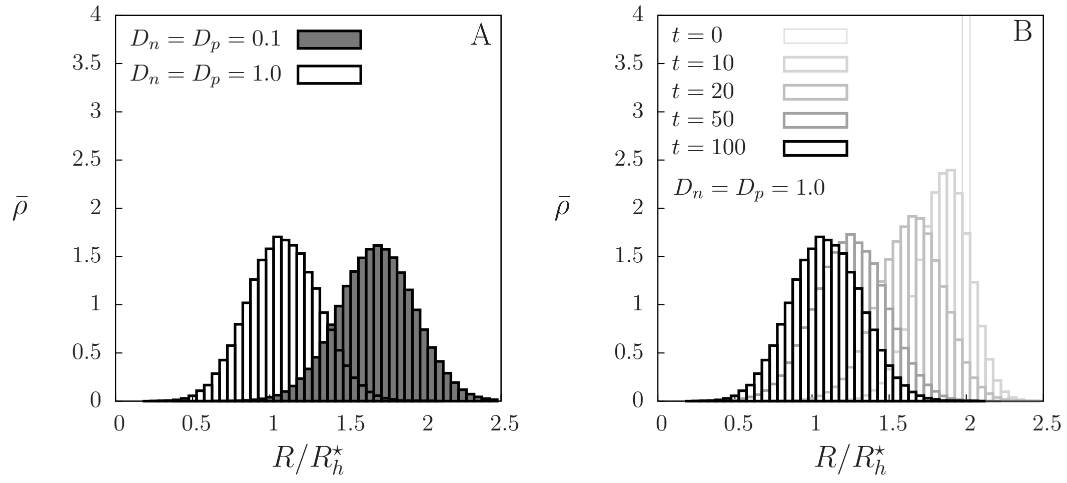

Figure S1: **Normalized perceptual-range probability density function at long times and its temporal evolution.** (A) $\bar{\rho}(R) = \rho(R)/N_p$ at long-times for low ($D_p = D_v = 0.1$) and high ($D_p = D_v = 1$) diffusion. (B) Starting from a delta distribution at $R = 4$, time evolution of $\bar{\rho}(R)$ obtained from the individual-level simulations with high diffusion $D_p = D_v = 1$. In both panels, the system size is $L = 10$ and mutation intensity $\sigma_\mu = 0.1$. In both panels, the values of the perceptual ranges are scaled by the optimal value $R_h^\star$ ($= 2R_c = 2$) of the homogeneous case for comparison.

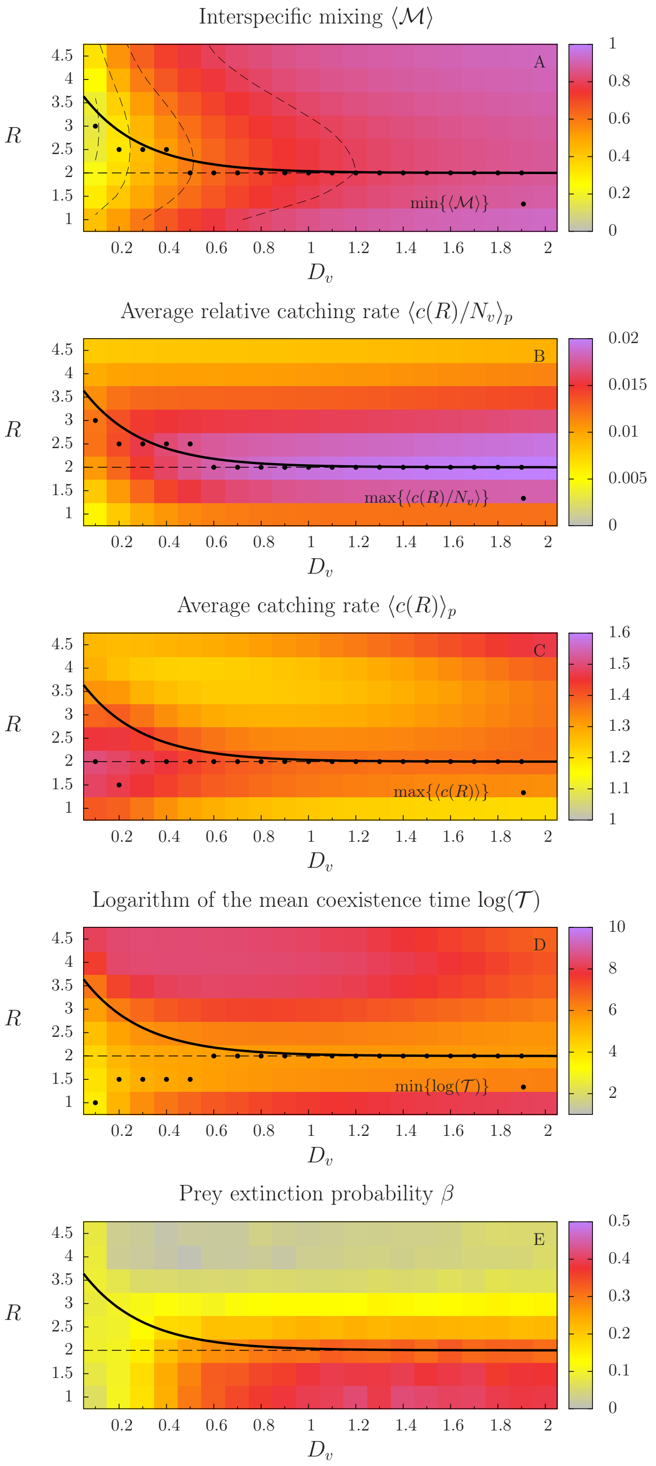

Figure S2: **Community-level properties as a function of predator perceptual range, R, and individual diffusion coefficients ($D_p = D_v$) in the absence of evolution.** From top to bottom: (A) average interspecific mixing, (B) average relative catching rate, (C) average catching rate, (D) average coexistence time (D), and (E) prey extinction probability. For all the cases, $L = 10$ and the perceptual range is the same for all predators and remains constant ($\sigma_\mu = 0$). The solid black line represents the curve $R^\star(D_v)$ selected by the evolutionary dynamics in the limit of very weak mutations. The dashed, horizontal line shows the optimal perceptual range $R_h^\star = 2R_c = 2$ in the well-mixed limit. Dots indicate maximal or minimal values, as indicated in the legend, when varying R at fixed D_v. In panel A, dashed curves are contour levels of $\langle \mathcal{M} \rangle$.

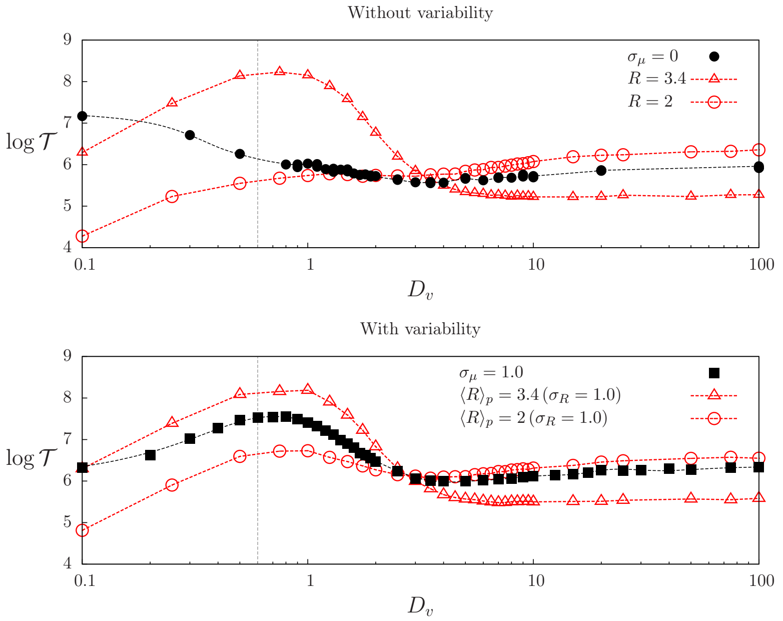

Figure S3: **Community coexistence times for different sources of variability in predator perceptual ranges.** Mean coexistence time $\mathcal{T}$ as a function of the diffusion coefficients $D_p = D_v$. Top panel: no variability in perceptual ranges. Black circles correspond to the no-mutation limit ($\sigma_\mu = 0$) also shown in Fig. 8 in the main text, red symbols and lines are from simulations in which all predator perceptual ranges take the values shown in the legend. Bottom panel: variability in predator perceptual ranges. The black line corresponds to the outcome of the evolutionary model with $\sigma_\mu = 1$, also displayed in Fig. 8. Red symbols and lines correspond to simulations in which the perceptual range of the predators is sampled from Normal distributions with mean $\langle R \rangle_p$ and standard deviation σ_R (values shown in the legend). These distributions are similar to the ones obtained from evolutionary simulations and shown in Fig. S1. Results were extracted from 5×10^3 realizations in which prey and predators were initially distributed uniformly in space. Dashed curves are smooth fits to guide the eye. Vertical dashed lines indicate the value of the diffusion coefficients at which the increase in mixing with respect to the no-mutation case is maximum (from Fig. 5, $\sigma_\mu = 1.0$).

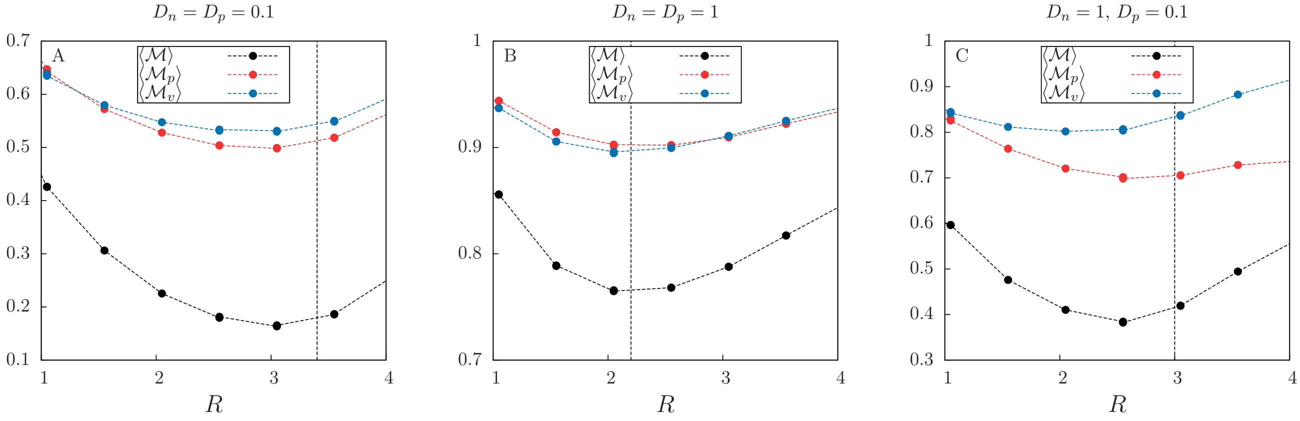

Figure S4: **Mixing measures as a function of predator perceptual range R in the non-evolutionary model.** Prey-predator, prey, and predator mixing for different levels of diffusion as a function of R (which remains constant, $\sigma_\mu = 0$, and is the same for all individuals). Vertical lines show the dominant perceptual range selected by the evolutionary dynamics under low mutation noise ($\sigma_\mu = 0.1$). For comparison, notice that the dominant perceptual range is $R_h^\star = 2R_c = 2$ in the homogeneous limit. Habitat size $L = 10$.

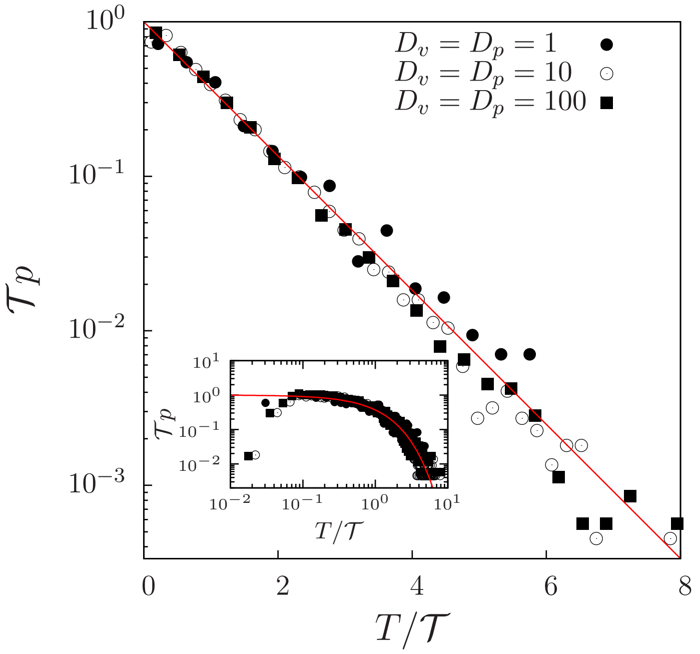

Figure S5: **Coexistence-time probability distribution.** Coexistence-time probability distribution p (shown multiplied by the mean value $\mathcal{T}$) obtained from individual-level simulations with $D_v = D_p = 1$, 10, 100, mutation intensity $\sigma_\mu = 1.0$ and habitat size $L = 10$. Inset shows the behavior at short timescales for the same cases. Solid red lines indicate the exponential distribution with the same mean, $p = \mathcal{T}^{-1}e^{-T/\mathcal{T}}$.

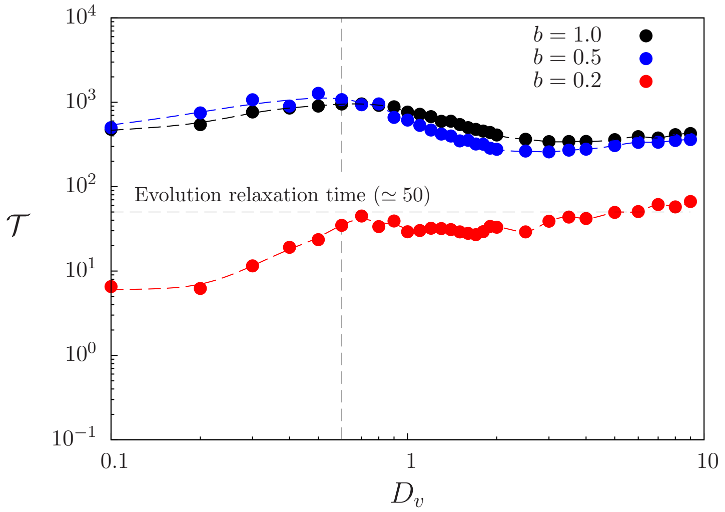

Figure S6: **Influence of predator birth probability b on coexistence times.** Mean coexistence time $\mathcal{T}$ as a function of the diffusion coefficients ($D_p = D_v$) for $\sigma_\mu = 0.5$ and system size $L = 10$. Different color curves account for different predator birth probabilities b. There is a range of values of b for which the behavior of $\mathcal{T}$ is robust and similar to the one discussed in the main text ($b = 1$). For b sufficiently small, however, the mean coexistence time is shorter than the evolutionary time scale, that is, the time needed by the eco-evolutionary feedback to reach the stationary distribution of perceptual ranges (horizontal, dashed line). In consequence, the behavior of $\mathcal{T}$ changes. In these simulations, we used and initial condition such that prey and predators are uniformly distributed in space and the perceptual ranges R are uniformly distributed in $[0, L/2]$. Averages are obtained over 10^3 realizations, dashed curves are smooth fits to simulation data to guide the eye. The vertical, dashed line indicates the value of the diffusion coefficient at which the increase of mixing with respect to the no-mutation case is maximum (see Fig. 5 in the main text).

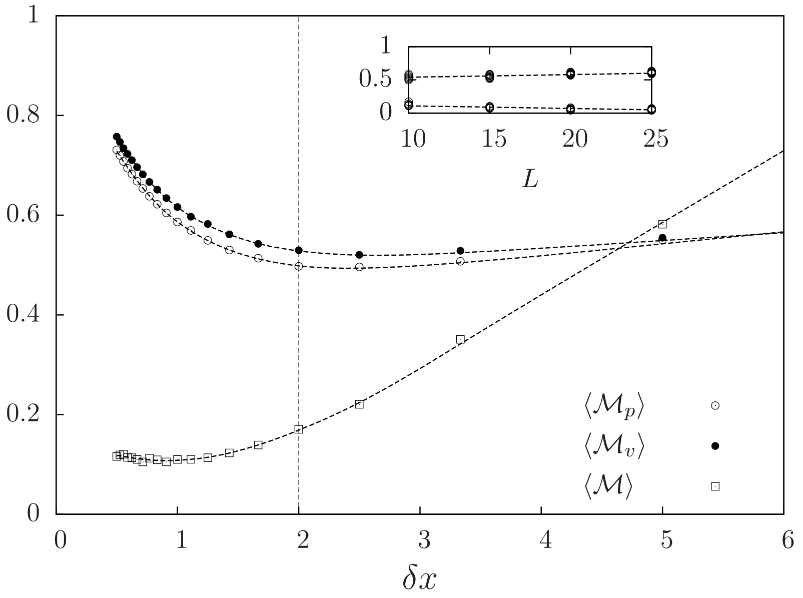

Figure S7: **Shannon entropy-based measures of population mixing as a function of the size of the cells used in their calculation.** Parameter values in the main panel: $D_p = D_v = 0.1$, mutation intensity $\sigma_\mu = 0.1$, and habitat size $L = 10$. In the inset, $\delta x = 2$ and varying L (for systems sizes that are not multiple of the cell size chosen, we choose the number of cells m such that $\delta x = L/m \simeq 2$).
